# Navigating Drug-Induced Lung Disease (DILD): A Comprehensive Review on Management and Prevention Strategies

**DOI:** 10.7759/cureus.69954

**Published:** 2024-09-22

**Authors:** Srinivasulareddy Annareddy, Babaji Ghewade, Ulhas Jadhav, Pankaj Wagh

**Affiliations:** 1 Respiratory Medicine, Jawaharlal Nehru Medical College, Datta Meghe Institute of Higher Education and Research, Wardha, IND

**Keywords:** adverse drug reactions, diagnostic evaluation, drug-induced lung disease (dild), management strategies, preventive measures, pulmonary toxicity

## Abstract

Drug-induced lung disease (DILD) is a significant and often overlooked adverse effect of pharmacological treatments, encompassing a range of pulmonary disorders triggered by medications. This review provides a comprehensive overview of DILD, focusing on its definition, pathophysiology, and clinical implications. We explore the epidemiology of DILD, highlighting the prevalence of various drugs associated with pulmonary toxicity and the factors influencing susceptibility. The review details the clinical presentation of DILD, including common symptoms and diagnostic challenges, and outlines diagnostic modalities such as imaging, pulmonary function tests, and invasive procedures. Management strategies are discussed, emphasizing the importance of timely drug discontinuation, supportive care, and the role of corticosteroids and novel therapies. Preventive measures, including pre-treatment evaluations and ongoing monitoring, are also addressed. The review concludes by examining future research directions and emerging therapies, aiming to enhance the understanding and management of DILD. This review is intended to aid healthcare professionals in recognizing, managing, and preventing drug-induced lung diseases, ultimately improving patient outcomes and safety.

## Introduction and background

Drug-induced lung disease (DILD) encompasses a broad spectrum of pulmonary disorders caused by various pharmacological agents. These diseases range from mild, reversible conditions to potentially fatal complications [1]. The term "DILD" refers to any adverse pulmonary effect directly attributable to the administration of a drug. These effects can be acute or chronic, affecting various respiratory system parts, including the airways, alveoli, interstitium, and pleura [2]. Understanding and managing DILD is crucial for several reasons. First, the clinical presentation of DILD can mimic other common pulmonary diseases, making diagnosis challenging. Misdiagnosis or delayed recognition of DILD can lead to unnecessary treatments, increased morbidity, and even mortality [3]. Second, as the number of available drugs grows, so does the potential for adverse pulmonary reactions. Healthcare providers must be vigilant and knowledgeable about the possible respiratory side effects of their medications. Third, prompt identification and management of DILD can significantly improve patient outcomes, often by discontinuing the offending drug and initiating appropriate therapy [2].

This comprehensive review aims to elucidate the complexities of drug-induced lung disease by thoroughly examining its epidemiology, pathophysiology, clinical presentation, and diagnostic evaluation. It will also cover common drugs associated with DILD and offer practical management strategies, including supportive care and pharmacologic interventions. Preventive measures and future research and clinical practice directions will also be discussed. Through this review, healthcare professionals will better understand DILD, enhancing their ability to effectively diagnose, manage, and prevent this challenging condition.

## Review

Epidemiology and incidence

Drug-induced lung disease (DILD) represents a significant concern in pharmacotherapy, with prevalence rates varying depending on the drug class involved [4]. Among different medication categories, antineoplastic agents are the most frequently implicated, accounting for approximately 47.69% of reported DILD cases. Notably, drugs such as bleomycin are well-known for their potential to cause pulmonary toxicity, with incidence rates reaching up to 10% in treated patients. Cardiovascular drugs also contribute to the prevalence of DILD, representing around 11.18% of cases, with amiodarone associated with a lung toxicity incidence of about 6% [5]. Antirheumatic agents, including methotrexate, are another significant contributor, with chronic toxicity occurring in approximately 7% of patients.

Other drug classes, such as antibiotics and anti-inflammatory medications, also play a role, highlighting the diverse range of pharmaceuticals that can lead to lung complications [6]. Several specific drugs have been frequently associated with DILD. Bleomycin stands out for its high incidence of pulmonary toxicity, affecting up to 10% of patients undergoing treatment. Amiodarone, known for its cardiovascular benefits, carries a 6% risk of lung toxicity, while methotrexate has a chronic toxicity incidence of around 7% [7]. Nitrofurantoin is another notable drug linked to acute lung toxicity in about one out of 5,000 cases. Additionally, other chemotherapeutic agents like busulfan, mitomycin, and cyclophosphamide contribute to the overall incidence of DILD, underscoring the importance of awareness among healthcare providers regarding these medications' potential pulmonary side effects [8].

Several factors can influence an individual's susceptibility to DILD, including age, comorbidities, and genetic predispositions. Age is a critical factor, as both older adults and children are at increased risk for drug toxicity. The elderly, in particular, may experience severe side effects due to diminished organ function and altered drug metabolism. Studies suggest that patients over 40 years old, especially those with compromised renal function, have a higher risk of experiencing fatal toxicity from certain medications. Comorbidities also play a significant role; patients with underlying conditions such as rheumatoid arthritis or inflammatory bowel disease are more likely to develop DILD, particularly when receiving multiple concurrent therapies that may be toxic to the lungs [9]. Genetic factors further complicate the landscape of DILD susceptibility. Genetic polymorphisms can significantly affect individual medication responses, especially in drug-metabolizing enzymes such as the cytochrome P450 family. Variations in these enzymes can lead to differences in drug efficacy and toxicity, suggesting that genetic testing may help identify patients at higher risk for DILD [10]. Additionally, there are indications of ethnic differences in the incidence of DILD, with certain populations showing higher susceptibility. For instance, specific cases of lung disease induced by gefitinib have been predominantly documented in Japanese patients, highlighting the potential for racial variations in drug response and toxicity [11]. Understanding these epidemiological factors is crucial for clinicians to anticipate, recognize, and effectively manage DILD. By being aware of the prevalence of DILD across different drug classes, the common drugs implicated, and the factors influencing susceptibility, healthcare providers can better protect patients from this serious complication of pharmacotherapy [12].

Pathophysiology

The pathophysiology of drug-induced lung injury (DILI) is complex, involving various mechanisms that can lead to pulmonary complications. These mechanisms can be broadly categorized into two main types: direct toxicity and immune-mediated toxicity [1]. Direct toxicity occurs when a drug or its metabolites cause cellular damage without involving the immune system. This can happen through several pathways, such as producing reactive metabolites, oxidative stress, and direct cellular injury. For example, drugs like bleomycin can generate reactive oxygen species (ROS), leading to oxidative stress, cell injury, and apoptosis. Additionally, certain drugs can directly damage alveolar epithelial cells or endothelial cells, causing inflammation and pulmonary edema [13]. Immune-mediated lung injury involves an abnormal immune response triggered by drug exposure. This can manifest through allergic reactions, where drugs act as haptens and bind to proteins, eliciting an immune response. This can result in drug-induced pneumonitis, characterized by the infiltration of immune cells and cytokine release. Some medications can also induce systemic cytokine release, causing lung inflammation and damage, such as capillary leakage and pulmonary edema [14]. Genetic factors significantly influence susceptibility to drug-induced lung injury. Variability in drug metabolism and immune response can lead to idiosyncratic reactions, which are unpredictable and not dose-dependent. Genetic polymorphisms in genes encoding drug-metabolizing enzymes can affect the production of toxic metabolites, while preexisting lung conditions or genetic predispositions may increase the risk of developing DILI. Differences in immune system function among individuals can also result in varying responses to drugs, with some patients experiencing severe immune-mediated lung injury while others do not [15]. Understanding the pathophysiology of drug-induced lung injury, including the influence of genetic predisposition and idiosyncratic reactions, is crucial for identifying at-risk patients and developing effective prevention and management strategies. Ongoing research is essential to elucidate these mechanisms further and improve clinical outcomes for patients affected by drug-induced lung injury [16].

Clinical presentation

**Figure 1:**
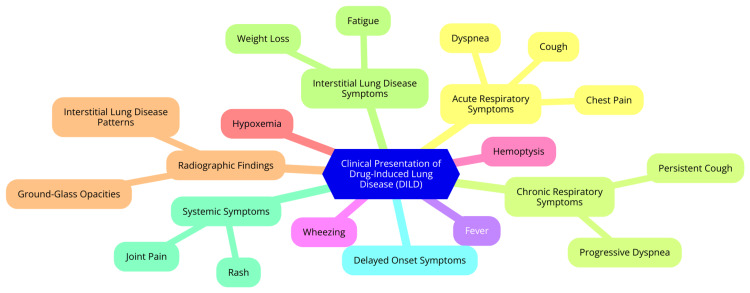
The clinical presentation of drug-induced lung disease (DILD) Image Credit: Dr Srinivasulareddy Annareddy

Drug-induced lung disease (DILD) encompasses a range of clinical manifestations, from acute respiratory distress to chronic pulmonary conditions. Understanding these manifestations, common symptoms, and the challenges in differential diagnosis is essential for effective management [1]. DILD can be categorized into acute and chronic presentations. Acute DILD often develops rapidly, sometimes within days of exposure to the offending drug. This can lead to conditions such as acute respiratory distress syndrome (ARDS), characterized by severe dyspnea, hypoxemia, and diffuse pulmonary infiltrates on imaging. Acute presentations may also include hypersensitivity reactions, manifesting as cough, fever, and eosinophilia [17]. Conversely, chronic DILD typically develops more insidiously, often presenting weeks to months after drug exposure. Patients may experience progressive dyspnea and cough, with potential long-term consequences such as interstitial lung disease or pulmonary fibrosis. Chronic symptoms can be subtle and may be mistaken for other chronic respiratory conditions [17]. The symptoms of DILD are often nonspecific and can vary widely depending on the underlying mechanism and specific drug involved. Common symptoms include dyspnea (shortness of breath, often worsening with exertion). This persistent dry cough may be accompanied by sputum production, fever (which may indicate a hypersensitivity reaction), pleuritic chest pain (particularly if pleural effusions are present), and crackles on auscultation (often described as "Velcro crackles"), especially in cases of interstitial lung disease [18]. Diagnosing DILD presents significant challenges due to the lack of pathognomonic signs and the overlap of symptoms with other pulmonary conditions. A key challenge is the temporal relationship between symptoms and drug exposure, as symptoms may not manifest until after the drug has been discontinued, complicating the identification of the offending agent. This latency can lead to misdiagnosis or delayed treatment. A thorough history and exclusion of other potential causes of lung disease (such as infections, autoimmune disorders, or environmental exposures) are essential, often requiring a systematic approach and advanced imaging and laboratory tests. Furthermore, many drugs can induce similar pulmonary syndromes, making it difficult to pinpoint the exact cause [19]. The clinical presentation of drug-induced lung disease (DILD) is shown in Figure 1.

Diagnostic evaluation

The diagnostic evaluation of drug-induced lung disease (DILD) is a comprehensive process that integrates various imaging modalities, pulmonary function tests (PFTs), and invasive procedures such as bronchoalveolar lavage (BAL) and lung biopsy. Each component is essential for accurately diagnosing and managing DILD, crucial for effective treatment, and improved patient outcomes [1]. Imaging modalities play a critical role in the initial assessment of DILD. Chest X-ray is typically the first imaging technique used, offering a preliminary evaluation of lung structures. It can identify gross abnormalities such as pleural effusions, large masses, or significant infiltrates. However, its sensitivity is limited, and subtle interstitial lung disease changes may not be detected [20]. In contrast, high-resolution computed tomography (HRCT) is considered the gold standard for evaluating diffuse infiltrative lung diseases, including DILD. HRCT provides superior detail compared to chest X-rays, allowing for visualization of the lung interstitium and identification of specific patterns associated with various forms of lung disease, such as ground-glass opacities, reticular patterns, and honeycombing. Studies have shown that HRCT significantly enhances diagnostic accuracy, with confidence levels reaching up to 93% for CT scans compared to 77% for chest X-rays [21]. Pulmonary function tests (PFTs) are essential for assessing lung function in patients suspected of having DILD. These tests measure various parameters, including forced vital capacity (FVC), forced expiratory volume in 1 second (FEV1), and diffusing capacity of the lung for carbon monoxide (DLCO). In DILD, PFTs often reveal a restrictive pattern characterized by reduced lung volumes and impaired gas exchange. This information is critical for differentiating DILD from other respiratory conditions and helps clinicians understand the extent of lung involvement [22]. Further evaluation through bronchoalveolar lavage (BAL) and lung biopsy may be necessary in some cases. BAL is a minimally invasive procedure involving instilling saline into a lung segment and retrieving it for analysis. This technique is valuable for obtaining cellular and biochemical information about the lung interstitium, which can help identify inflammatory or infectious processes. In the context of DILD, BAL may reveal lymphocytic predominance or other cellular patterns indicative of specific drug reactions. When the diagnosis remains unclear after non-invasive evaluations, lung biopsy-either via transbronchial or open surgical methods, be warranted. Histopathological examination of lung tissue can provide definitive evidence of drug-induced lung injury, allowing for the classification of the underlying pathology, such as interstitial pneumonia or granulomatous inflammation [23].

Common drugs associated with DILD

**Figure 2:**
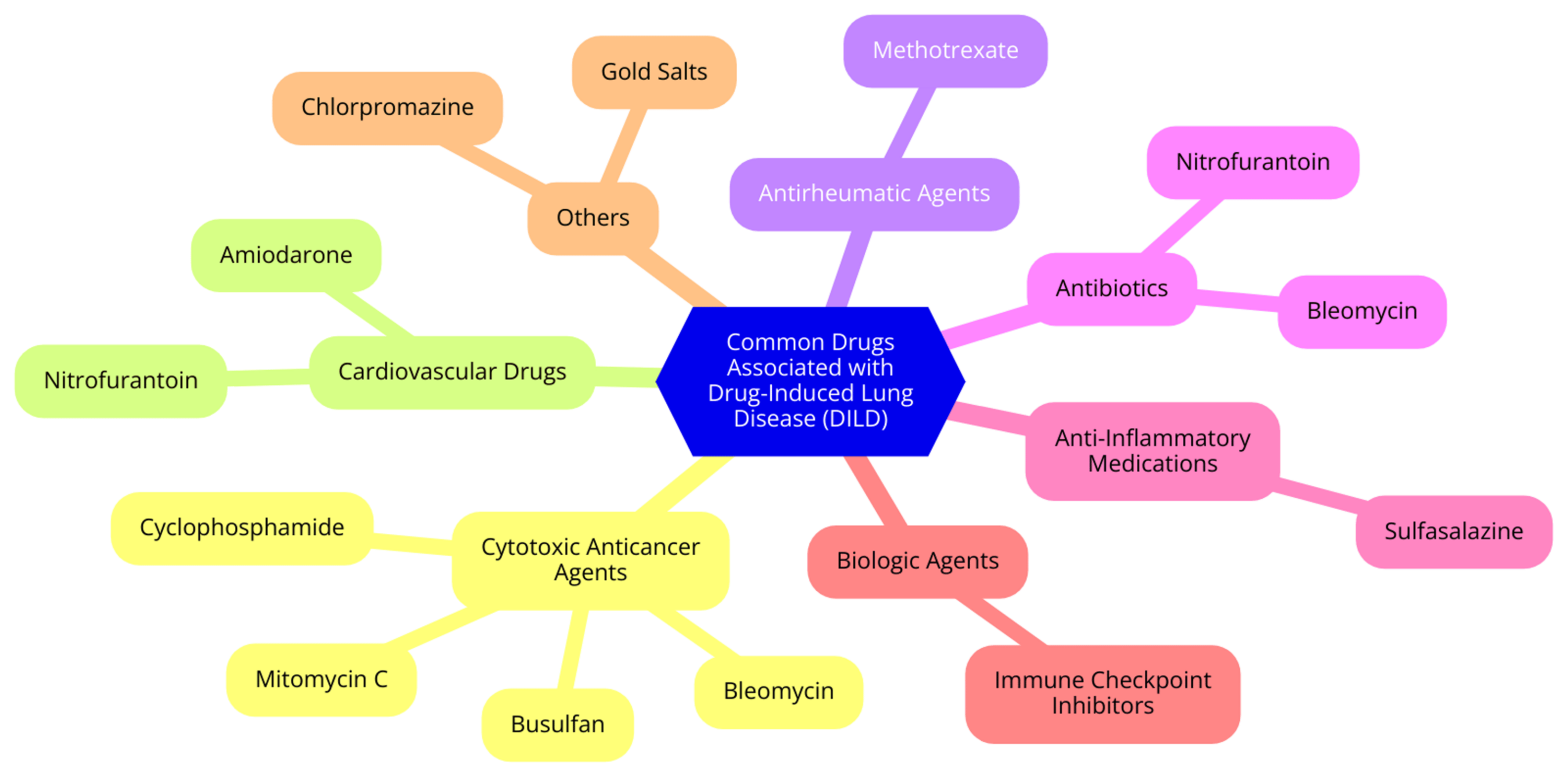
Common drugs associated with drug-induced lung disease (DILD) Image Credit: Dr Srinivasulareddy Annareddy

Drug-induced lung disease (DILD) can result from a diverse array of medications across various therapeutic classes. Recognizing the specific drugs associated with DILD is crucial for clinicians to monitor and manage potential respiratory complications [24] effectively. Antibiotics are frequent contributors to DILD. Nitrofurantoin, in particular, is well-known for causing pulmonary toxicity, especially with long-term use. Patients may experience symptoms ranging from cough and dyspnea to more severe pulmonary reactions. Sulfasalazine is another antibiotic associated with hypersensitivity reactions that can lead to lung complications, including interstitial lung disease [25]. Chemotherapeutic agents are also significant contributors to DILD. Bleomycin is often linked to interstitial lung disease, especially in cancer patients, with a reported incidence of up to 10%. The risk is elevated in individuals receiving high cumulative doses or those with pre-existing lung conditions. Methotrexate, used for various malignancies and autoimmune disorders, can also cause pneumonitis and other lung complications, particularly in patients with a history of lung disease [26]. In the category of immunosuppressants, methotrexate again emerges as a notable agent associated with lung injury. Its immunosuppressive effects can predispose patients to pulmonary complications. Additionally, TNF inhibitors such as infliximab and adalimumab are known to cause lung issues, including interstitial lung disease. While these medications effectively treat conditions like rheumatoid arthritis and inflammatory bowel disease, they require vigilant monitoring for respiratory symptoms [27]. Cardiovascular drugs can also induce DILD. Amiodarone, a well-known antiarrhythmic medication, is associated with pulmonary toxicity, which can manifest as interstitial pneumonitis or fibrosis. The risk of lung injury increases with prolonged use and higher doses. Although less common, ACE inhibitors can lead to cough and angioedema, complicating the management of patients with pre-existing respiratory conditions [28]. Common Drugs Associated with Drug-Induced Lung Disease (DILD) are shown in Figure 2.

Management strategies

Effective management of drug-induced lung disease (DILD) requires a comprehensive approach that includes immediate cessation of the offending drug, supportive care, pharmacological interventions, and exploration of novel therapies. Each strategy is critical in achieving optimal patient outcomes [29]. Immediate discontinuation of the offending drug is the cornerstone of DILD management. This strategy often leads to rapid symptom relief, typically within 24 to 48 hours in acute cases. The primary goal of immediate cessation is to prevent further lung damage and facilitate recovery. In certain situations, gradual withdrawal may be necessary for medications that cause dependence or withdrawal symptoms. However, this is generally not recommended for DILD, as tapering can exacerbate pulmonary symptoms. When withdrawal symptoms are anticipated, a carefully monitored tapering schedule may be used, but this approach is less common in DILD management [30]. Supportive care is an essential component of DILD treatment. Key aspects include smoking cessation, which is crucial for enhancing lung health and preventing additional damage. Managing underlying lung conditions, such as chronic obstructive pulmonary disease (COPD) or asthma, is vital for improving patient outcomes. Prompt treatment of respiratory infections is necessary, as these infections can complicate the clinical picture and worsen respiratory symptoms. Addressing these factors can significantly aid recovery and improve the overall prognosis for patients with DILD [31]. Corticosteroids are frequently used in treating DILD, especially in cases of severe lung toxicity or persistent symptoms despite the discontinuation of the offending drug. These medications can lead to rapid improvements in gas exchange and may help reverse radiographic abnormalities associated with cryptogenic organizing pneumonia and drug-induced eosinophilic pneumonia. If corticosteroids are insufficient or contraindicated, immunosuppressive therapies may be considered. Agents such as azathioprine or mycophenolate mofetil can be effective in chronic or progressive cases of DILD where inflammation persists despite initial treatment efforts [1]. In addition to traditional treatments, emerging therapies, particularly biologics, are being investigated for their potential role in managing DILD. These therapies target specific pathways involved in the inflammatory response and may offer benefits in cases where conventional treatments fail. For instance, agents that inhibit immune checkpoints or modulate cytokine responses are currently under investigation and could offer new treatment options for drug-induced interstitial lung disease. Ongoing research in this area holds promise for expanding treatment options and improving outcomes for patients with DILD [32].

Preventive measures

Preventing drug-induced lung disease (DILD) necessitates a comprehensive strategy involving proactive screening, vigilant monitoring during treatment, and thorough patient education. Each element is crucial in reducing the risk of medication-associated pulmonary complications [2]. Screening begins with a detailed medical history. Healthcare providers should gather extensive information about the patient's past drug reactions, respiratory conditions, and known allergies. This data is essential for identifying individuals at higher risk for DILD. Additionally, validated risk assessment tools can help evaluate the likelihood of lung toxicity linked to specific medications. These tools typically consider factors such as age, pre-existing lung conditions, and concurrent medications, allowing for a more customized approach to patient care [33]. Before initiating treatment with potentially harmful drugs, baseline pulmonary function tests (PFTs) should be conducted. Establishing a reference point for lung function is particularly important for patients starting high-risk medications, such as chemotherapeutics or biologics. In some instances, baseline chest imaging, such as a chest X-ray or CT scan, may also be necessary to assess the lung condition before treatment begins [22]. During treatment, regular follow-up appointments are crucial for monitoring respiratory health. Frequent visits, especially during the initial stages of therapy, facilitate early detection of any adverse effects. Periodic pulmonary function testing should be performed to monitor changes in lung function throughout the treatment course. This is especially critical for drugs known to have pulmonary side effects [34]. Encouraging patients to monitor their symptoms is also a vital preventive measure. Patients should be advised to report any new or worsening respiratory symptoms, such as cough, dyspnea, or chest pain. Implementing a symptom diary can help patients track changes and communicate effectively with their healthcare providers. Follow-up imaging studies may be necessary if respiratory symptoms develop or significant changes in PFT results are observed, enabling early differentiation between drug-induced changes and other pulmonary conditions [35].

Patient education is a cornerstone of preventing DILD. Healthcare providers should inform patients about their medications' potential pulmonary side effects, enabling them to recognize symptoms early. It is crucial to educate patients on how to monitor their respiratory health and recognize early signs of lung toxicity. This education should include guidance on when to seek medical attention, ensuring that patients can manage their health proactively [36]. Promoting smoking cessation is another important preventive strategy, particularly for patients who smoke, as smoking can increase the risk of lung complications. Providing resources and support for quitting can significantly improve patient outcomes. Distributing written materials, such as brochures or handouts detailing the signs and symptoms of DILD, can reinforce verbal education and serve as a valuable reference for patients [37]. Involving family members or caregivers in the education process is also beneficial. They can assist in monitoring the patient’s condition and support adherence to treatment and follow-up appointments, creating a more comprehensive support system for the patient [38].

Prognosis and outcomes

Several key factors influence the prognosis of drug-induced lung disease (DILD). One of the primary determinants is the type of medication involved. For example, cytotoxic anticancer agents are associated with a significantly higher mortality rate compared to targeted therapies such as kinase inhibitors (KIs) or immune checkpoint inhibitors (ICIs) [39]. Research indicates that the 90-day mortality rate for patients treated with cytotoxic agents can be around 30%, a stark contrast to the outcomes seen with KIs or ICIs. Additionally, the severity of DILD at the time of diagnosis is a crucial prognostic indicator. Patients with severe manifestations often face worse outcomes, including higher rates of respiratory failure and treatment-related mortality. Pre-existing conditions also play a significant role; individuals with a history of interstitial lung disease (ILD) or a performance status (PS) of 2-4 generally have a poorer prognosis, complicating their clinical management and recovery [40]. Long-term outcomes for patients with DILD can vary widely based on the initial severity of the disease and the specific medication involved. Although many patients may see improvement after discontinuing the offending drug, some may experience persistent chronic symptoms, especially if pulmonary fibrosis develops. This chronicity can lead to long-term respiratory issues, with some patients failing to recover their lung function fully. Mortality rates associated with DILD can be substantial, particularly among those who have received aggressive cancer therapies. Studies show that overall mortality related to DILD is significant, particularly in severe cases, underscoring the need for vigilant monitoring and management [1]. The impact of DILD on a patient's quality of life and functional status can be profound. Many individuals experience a decline in their ability to perform daily activities due to respiratory symptoms, leading to increased dependence on caregivers and a reduced overall quality of life. Psychosocial effects often accompany this decline; the chronic nature of the symptoms and the potential for long-term health issues can contribute to feelings of anxiety and depression. Consequently, patients may require comprehensive management strategies that address their health's physical and psychological aspects. Effective support and rehabilitation programs are essential for helping patients regain functional capacity and improve their quality of life following a diagnosis of DILD [41].

Future directions and research needs

Despite notable advancements in understanding and managing drug-induced lung disease (DILD), several areas warrant further research to improve patient outcomes. One critical focus is deepening our understanding of the mechanisms underlying DILD. Investigating the pathways and genetic factors contributing to DILD development is crucial for identifying high-risk patients and creating targeted preventive strategies. Additionally, exploring the role of immune dysregulation and inflammation in the pathogenesis of DILD, especially with drugs like immune checkpoint inhibitors, could lead to more effective therapeutic interventions [1]. Another promising research direction is the integration of pharmacogenomics and personalized medicine into clinical practice. Pharmacogenomic testing has the potential to predict an individual's risk of developing DILD based on their genetic profile, enabling more customized treatment plans. Identifying biomarkers that can detect early lung injury and guide treatment decisions is also an active area of investigation. Moreover, developing personalized dosing algorithms that consider patient characteristics and genetic factors could help minimize the risk of DILD while ensuring therapeutic efficacy [42]. Emerging therapies and preventive strategies are crucial areas for future research in DILD. Novel anti-fibrotic agents and targeted immunomodulatory therapies are being explored as potential treatments for established DILD, offering hope for improved patient outcomes. Preventive measures, such as early detection of lung injury, dose optimization, and combination therapies, may significantly reduce the incidence of DILD. Enhancing education and awareness among healthcare providers about the risks and management of DILD is also essential for timely diagnosis and intervention [43].

## Conclusions

In conclusion, drug-induced lung disease (DILD) presents a significant challenge in clinical practice due to its diverse manifestations and potential for severe outcomes. A comprehensive understanding of DILD-its epidemiology, pathophysiology, and clinical presentation-is essential for accurate diagnosis and effective management. Early identification of the offending drug and appropriate therapeutic interventions can significantly improve patient outcomes and prevent long-term complications. Implementing preventive measures, such as pre-treatment screening and vigilant monitoring, is crucial in mitigating the risks associated with DILD. As our knowledge of drug-induced pulmonary disorders advances, ongoing research and innovations in drug safety, diagnostic techniques, and treatment strategies will enhance patient care. By staying informed and proactive, healthcare professionals can better navigate the complexities of DILD, ultimately improving patient safety and quality of life.
